# The Association Between Adult Height and Stroke Incidence in Japanese Men and Women: A Population-based Case-Control Study

**DOI:** 10.2188/jea.JE20200531

**Published:** 2023-01-05

**Authors:** Yoshinobu Kondo, Hiroshi Yatsuya, Atsuhiko Ota, Shoji Matsumoto, Akihiro Ueda, Hirohisa Watanabe, Hideaki Toyoshima

**Affiliations:** 1 Bureau of Health and Medical Care, Aichi Prefectural Government, Aichi, Japan; 2 Department of Public Health and Health Systems, Nagoya University Graduate School of Medicine, Aichi, Japan; 3 Department of Public Health, Fujita Health University School of Medicine, Aichi, Japan; 4 Department of Comprehensive Strokology, Fujita Health University School of Medicine, Aichi, Japan; 5 Department of Neurology and Neuroscience, Fujita Health University School of Medicine, Aichi, Japan; 6 Nagoya University Graduate School of Medicine, Aichi, Japan

**Keywords:** adult height, stroke incidence, ischemic stroke subtypes, case-control study, Japanese men and women

## Abstract

**Background:**

No studies have examined the associations between adult height and ischemic stroke subtypes.

**Methods:**

We conducted a population-based case-control study that included 2,451 thrombotic and 687 embolic stroke cases, as well as 1,623 intracerebral and 768 subarachnoid hemorrhage cases without history of stroke aged 40–79 years, and the same number of sex- and age-matched controls. Cases and controls were grouped according to the quintile cut-off values of height in controls, and the third quintile, which was approximately the average height group, was used as the reference group. Height divided by one standard deviation of height in controls was also examined as a continuous variable. The analyses were carried out separately for participants aged 40–59 years and 60–79 years.

**Results:**

In both younger and older men, height was linearly inversely associated with total and thrombotic strokes, and the shortest quintile compared to the reference group was associated with increased risks of these strokes. Although height was linearly inversely associated with embolic stroke and intracerebral hemorrhage in younger men, the shortest quintile did not show increased risks of these strokes. Height did not seem to be associated with total stroke and any stroke subtypes in younger women. In contrast, the tallest quintile was significantly associated with increased risks of total stroke and intracerebral hemorrhage, and height tended to be positively associated with these strokes in older women.

**Conclusion:**

We reported the associations between adult height and ischemic stroke subtypes for the first time, which differed according to sex and age group.

## INTRODUCTION

Although the age-adjusted mortality rate of stroke in Japan was much higher than that in Western countries in the 1960s, the age-adjusted mortality and incidence rates of stroke started to decline dramatically in the 1970s.^1^ It is believed that changes in lifestyle and improvement in prevention and management of high blood pressure contributed to the decline.^2^ At the same time, case-mix of stroke changed significantly during the same period. Namely, the incidences of ischemic stroke, especially lacunar infarction and intracerebral hemorrhage, declined from the 1960s to the 2000s, while the incidences of atherothrombotic and cardioembolic infarction did not change.^3^^,^^4^

On the other hand, the average height of Japanese people increased simultaneously during the period,^5^ which might be related to the change of stroke and ischemic stroke case-mix.

There are already quite a few studies that have reported inverse associations between adult height and stroke incidence or mortality.^6^^–^^11^ However, discrepancies exist in the previous literature by sex, stroke subtype, and age group.^12^^–^^17^ Furthermore, there are even studies that did not find any associations between adult height and stroke.^18^^,^^19^

To our knowledge, only a few studies have examined the associations between adult height and stroke subtypes, and there are no studies that have examined the associations between adult height and ischemic stroke subtypes. The aim of this study was to examine the associations between adult height and incidence of total stroke, thrombotic stroke, embolic stroke, intracerebral hemorrhage, and subarachnoid hemorrhage according to sex in Japanese men and women.

## METHODS

### The Aichi Prefecture Cardiovascular Disease Registry

The Aichi Prefectural Government implemented the Aichi Prefecture Cardiovascular Disease Registry between 2001 and 2009. When doctors made the diagnosis of stroke (including thrombotic stroke, embolic stroke, intracerebral hemorrhage, and subarachnoid hemorrhage) or acute myocardial infarction, they registered it, as well as whether it was first or recurrent event, the patient’s age, height, weight, lifestyles (including smoking, alcohol consumption, and exercise), disease histories (including hypertension, hyperlipidemia, and diabetes), and diagnostic methods (computed tomography [CT], magnetic resonance imaging [MRI], autopsy, cerebral angiography, coronary arteriography, electrocardiogram, echocardiography, and others), using officially standardized registration forms defined in the Guideline for the Aichi Prefecture Cardiovascular Disease Registry.

### Definition of stroke

Occurrences of stroke subtypes were confirmed according to the following criteria. Ischemic stroke was to have a sudden and rapid onset of neurological deficits and an evidence of infarction ascertained using CT, MRI, or autopsy. Embolic stroke was defined as ischemic stroke with the evidence of embolus origins, such as atrial fibrillation (Af), valvular heart diseases, and acute myocardial infarction within three months. In contrast, thrombotic stroke was ischemic stroke that did not have the proof of embolus origins. Thrombotic stroke was not classified into lacunar and atherothrombotic infarction in the present study.

Intracerebral hemorrhage was to have a sudden and rapid onset of neurological deficits and an evidence of hemorrhage confirmed using CT, MRI, or autopsy. Subarachnoid hemorrhage was to have a sudden and rapid onset of symptoms, such as headache or decreased level of consciousness, with signs of meningeal irritation, including nuchal rigidity, Kernig’s sign, and Brudzinski’s sign. Subarachnoid hemorrhage usually has no local neurological deficits. Imaging and other investigations must confirm at least one of the following criteria: (1) hemorrhage at the Sylvian fissure, frontal lobe, basilar cistern, or ventricle using CT; (2) bloody or yellow cerebrospinal fluid and either aneurysm or arteriovenous malformation using cerebral angiography; (3) an autopsy confirmation of recent subarachnoid hemorrhage and either aneurysm or arteriovenous malformation. Autopsy or CT excluded the possibility of intracerebral hemorrhage. Cryptogenic stroke or stroke of undetermined cause was excluded from the analyses.

### Cases

A total of 5,529 first-stroke cases aged 40–79 years registered between 2001 and 2009 were analyzed in the present study (3,003 men, 2,526 women) (Table 1). Of 3,003 male cases, there were 1,443 thrombotic stroke cases (aged 40–59 years: 677; aged 60–79 years: 766), 387 embolic stroke cases (aged 40–59 years: 157; aged 60–79 years: 230), 895 intracerebral hemorrhage cases (aged 40–59 years: 473; aged 60–79 years: 422), and 278 subarachnoid hemorrhage cases (aged 40–59 years: 191; aged 60–79 years: 87). Of 2,526 female cases, there were 1,008 thrombotic stroke cases (aged 40–59 years: 377; aged 60–79 years: 631), 300 embolic stroke cases (aged 40–59 years: 86; aged 60–79 years: 214), 728 intracerebral hemorrhage cases (aged 40–59 years: 335; aged 60–79 years: 393), and 490 subarachnoid hemorrhage cases (aged 40–59 years: 278; aged 60–79 years: 212).

**Table 1.** The number of cases included in the present study

		Men	Women
40–59 years	Total stroke	1,498	1,076
	Thrombotic stroke	677	377
	Embolic stroke	157	86
	Intracerebral hemorrhage	473	335
	Subarachnoid hemorrhage	191	278

60–79 years	Total stroke	1,505	1,450
	Thrombotic stroke	766	631
	Embolic stroke	230	214
	Intracerebral hemorrhage	422	393
	Subarachnoid hemorrhage	87	212

### Controls

We used population-based controls. Briefly, they were randomly selected from the sex- and five-year age-group-matched participants in the Aichi Prefecture Health, Lifestyle, and Behavior Survey carried out by the Aichi Prefectural Government in 2001, 2004, 2009, and 2012. These surveys were originally conducted to set health goals and check if these goals have been achieved in the Health Japan 21 Aichi Plan, which was established in 2001. Inhabitants of Aichi Prefecture were randomly selected at a sampling rate of 1/1,000, and asked to respond to questions in an anonymous self-reported questionnaire. The response rates of the surveys were about 40–60%. To select control subjects, we matched cases on sex and age and classified them into eight groups: (1) aged 40–44 years, (2) aged 45–49 years, (3) aged 50–54 years, (4) aged 55–59 years, (5) aged 60–64 years, (6) aged 65–69 years, (7) aged 70–74 years, and (8) aged 75–79 years. Control subjects had no history of stroke.

### Main exposure variable

To examine the associations between height and the risk of stroke incidence, we divided controls into height quintiles. Cases were also divided into five groups according to the same cut-off values used in the controls. Groups with height of 165.0–167.9 cm in men and 152.3–155.1 cm in women (the third quintile) were used as the reference groups.

Height and weight of cases were registered by doctors, whereas those of controls were self-reported. Regarding the registration of height of cases, we conducted a survey within the present study on how specifically the doctors would obtain the information. As the result, height of cases would be obtained from the patient chart, which was found to be filled in by nurses with mostly self-reported or family-reported ones. However, there were differences in the proportions of self-report versus others-report according to stroke subtypes and age of the patients (eMaterials 1). Nevertheless, the proportions of self-report did not differ between men and women, specifically in non-thrombotic stroke cases or in older patients. Although height of both cases and controls was mostly self-reported, which had been reportedly generally valid but containing similar source of errors and biases,^20^^,^^21^ we carried out a sensitivity analysis using height of controls 1 cm shorter than originally self-reported in an attempt to address a possibility that the observed association might have been due to overestimated height of controls. Since the distributions of height differed according to age group, another sensitivity analysis was conducted using age-group-specific height quintile cut-off values of controls.

### Potential confounding variables

Smoking status was assessed as current, ex-, and never-smoking. Drinking habit was assessed as current, ex-, and non-drinking, regardless of frequency. Exercise habit was assessed as do or do not. Unknown status was assigned to those without valid information on smoking, drinking, and exercise habits. These lifestyles and medical histories of hypertension, hyperlipidemia, and diabetes were registered by doctors in cases, and were self-reported in controls. Regarding lifestyle and medical history information registered by doctors, we conducted an interview survey to doctors who specialize in the care of stroke patients on how specifically they would be obtained. We found that doctors would transcribe lifestyle information from the patient chart, which was filled in by nurses who interviewed the patients or their family. In terms of medical histories, doctors would also use medication information and test results.

### Statistical analyses

All statistical analyses were performed using SPSS software, version 24.0 for Windows (IBM Corp., Armonk, NY, USA). A *P*-value <0.05 using a two-tailed test was considered statistically significant. The age-adjusted means or percentages of body weight, body mass index (BMI; calculated from registered height and weight in cases and self-reported height and weight in controls), current smoking, current drinking, exercise, hypertension, hyperlipidemia, and diabetes were calculated using a general linear model according to the height quintiles. To evaluate the associations between height and stroke incidence, we calculated the odds ratios (ORs) and 95% confidence intervals (CIs) of height groups using conditional multivariable logistic regression analysis matched for age and adjusted for continuous BMI, smoking, drinking, exercise, hypertension, hyperlipidemia, and diabetes. Unknown lifestyle categories were included using dummy variables. Height divided by one standard deviation of height in controls was also examined as a continuous variable. Finally, interactions of age group with stroke by continuous height were examined using the Wald test.

### Ethical approval

This study was approved by Fujita Health University Ethics Review Committee (approval number: HM19-023).

## RESULTS

The mean height of cases was 165.6 cm in younger (aged 40–59 years) and 162.5 cm in older (aged 60–79 years) men, and 155.1 cm in younger and 152.7 cm in older women (Table 2). The mean height of controls was 168.2 cm in younger and 164.4 cm in older men, and 155.7 cm in younger and 152.1 cm in older women. Age was inversely associated with height in both cases and controls in younger men and younger women, as well as in older men and older women (eTable 1 and eTable 2). The proportions of current smoking seemed higher in the taller height quintiles of cases, but not so in controls in both men and women. The proportions of current drinking were also positively associated with height in cases and controls in both men and women. Distributions of exercise and disease histories did not differ according to height, though the proportions of hypertension seemed higher in the shorter height quintiles and those of hyperlipidemia seemed higher in the taller height quintiles in controls in older women.

**Table 2.** Potential confounding factors for stroke in total stroke cases and controls

		Men	Women
40–59 years, Cases	Age, years	50.9 (5.5)	52.4 (5.2)
	Height, cm	165.6 (8.4)	155.1 (6.5)
	Weight, kg	66.7 (13.8)	55.7 (10.5)
	Body mass index, kg/m^2^	24.2 (4.2)	23.1 (4.0)
	Current smoking, %	49.4 (1.3)	25.2 (1.1)
	Current drinking, %	47.7 (1.3)	25.1 (1.3)
	Exercise +, %	14.7 (1.0)	15.4 (1.3)
	Hypertension, %	56.5 (1.1)	53.4 (1.3)
	Hyperlipidemia, %	19.7 (0.9)	17.2 (1.0)
	Diabetes, %	21.6 (0.9)	16.0 (0.9)
40–59 years, Controls	Age, years	50.7 (5.5)	52.1 (5.3)
	Height, cm	168.2 (5.8)	155.7 (5.5)
	Weight, kg	66.0 (9.9)	53.5 (8.4)
	Body mass index, kg/m^2^	23.3 (3.1)	22.1 (3.4)
	Current smoking, %	34.4 (1.3)	8.4 (1.1)
	Current drinking, %	63.0 (1.3)	27.8 (1.3)
	Exercise +, %	27.8 (1.0)	33.7 (1.3)
	Hypertension, %	16.0 (1.1)	13.0 (1.3)
	Hyperlipidemia, %	11.2 (0.9)	8.4 (1.0)
	Diabetes, %	6.3 (0.9)	3.0 (0.9)
60–79 years, Cases	Age, years	67.7 (5.4)	68.0 (5.5)
	Height, cm	162.5 (7.7)	152.7 (7.3)
	Weight, kg	60.2 (11.0)	53.4 (10.7)
	Body mass index, kg/m^2^	22.7 (3.4)	22.9 (4.0)
	Current smoking, %	34.7 (1.1)	14.1 (0.7)
	Current drinking, %	42.9 (1.3)	14.3 (0.9)
	Exercise +, %	17.7 (1.1)	17.6 (1.2)
	Hypertension, %	62.1 (1.2)	61.0 (1.3)
	Hyperlipidemia, %	19.5 (0.9)	24.7 (1.0)
	Diabetes, %	25.8 (1.0)	20.6 (0.9)
60–79 years, Controls	Age, years	67.7 (5.3)	67.8 (5.3)
	Height, cm	164.4 (6.0)	152.1 (5.2)
	Weight, kg	62.1 (9.5)	51.7 (8.1)
	Body mass index, kg/m^2^	22.9 (3.1)	22.4 (3.3)
	Current smoking, %	21.1 (1.1)	4.2 (0.7)
	Current drinking, %	55.8 (1.3)	14.5 (0.9)
	Exercise +, %	48.2 (1.1)	46.6 (1.2)
	Hypertension, %	34.2 (1.2)	33.2 (1.3)
	Hyperlipidemia, %	10.6 (0.9)	15.0 (1.0)
	Diabetes, %	15.5 (1.0)	7.7 (0.9)

**Figure 1.**
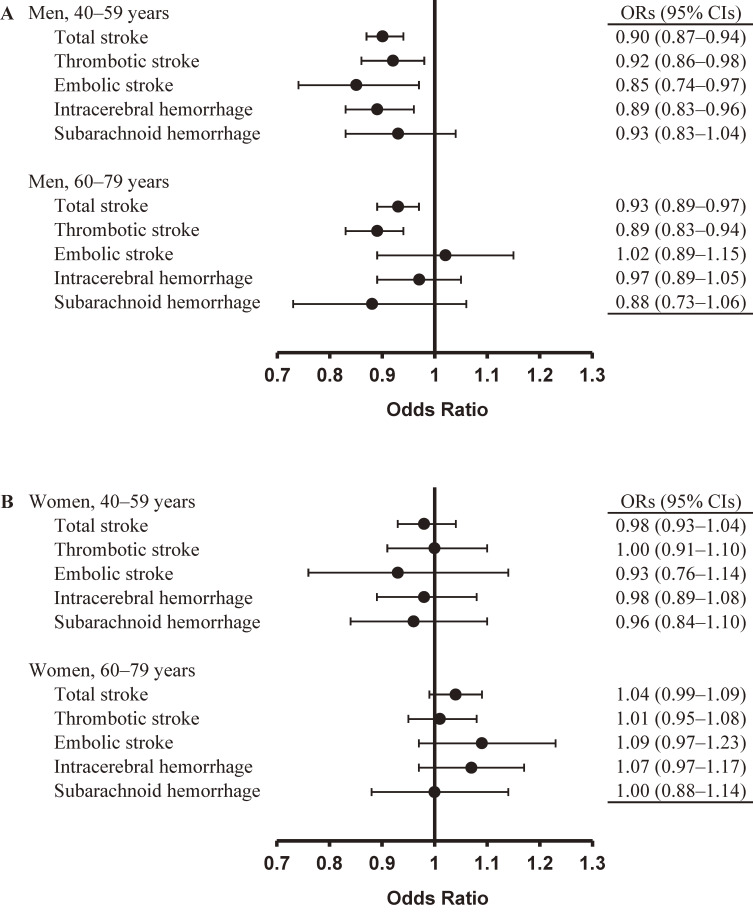
Multivariable-adjusted odds ratios and 95% confidence intervals of stroke and its subtypes for one standard deviation increase of height in men (A) and women (B). Odds ratios were matched for age and adjusted for body mass index, smoking, drinking, exercise, hypertension, hyperlipidemia and diabetes; OR, odds ratio; CI, confidence interval.

Compared to those with 165.0–167.9 cm height, younger men with height less than 161.0 cm (shortest quintile) had significantly higher risks of total and thrombotic strokes (model 2 ORs 1.34 and 1.40, respectively; Table 3A). In addition, continuous height was linearly and inversely associated with the risks of total, thrombotic, and embolic strokes, and intracerebral hemorrhage in younger men (ORs 0.90, 0.92, 0.85, and 0.89, respectively; Figure 1A). In older men, continuous height was linearly and inversely associated with the risks of total and thrombotic strokes (ORs 0.93 and 0.89, respectively), and the shortest height quintile tended to be associated with increased risks of total and thrombotic strokes (Table 3B). The association between height and embolic stroke seemed to differ by age group (*P* for interaction of age group with embolic stroke = 0.054).

**Table 3A.** Multivariable-adjusted odds ratios and 95% confidence intervals of stroke and its subtypes according to height in men aged 40–59 years, Aichi, Japan

		Height, cm				

		≤160.9	161.0–164.9	165.0–167.9	168.0–170.9	≥171.0
Total stroke	Case	368	178	239	318	395
	Control	150	236	282	365	465
	Model 1	1.55 (1.32–1.83)^a^	0.94 (0.77–1.14)	1	1.01 (0.86–1.20)	1.00 (0.85–1.17)
	Model 2	1.34 (1.13–1.58)^a^	0.97 (0.79–1.17)	1	0.98 (0.83–1.16)	0.99 (0.84–1.16)
Thrombotic stroke	Case	168	83	116	136	174
	Control	65	124	135	158	195
	Model 1	1.56 (1.23–1.98)^a^	0.87 (0.66–1.15)	1	1.00 (0.78–1.28)	1.01 (0.80–1.29)
	Model 2	1.40 (1.10–1.79)^b^	0.94 (0.71–1.25)	1	1.00 (0.78–1.28)	1.07 (0.85–1.36)
Embolic stroke	Case	43	16	22	35	41
	Control	18	24	27	37	51
	Model 1	1.57 (0.94–2.63)	0.89 (0.47–1.70)	1	1.08 (0.64–1.85)	0.99 (0.59–1.66)
	Model 2	1.36 (0.79–2.34)	0.86 (0.44–1.67)	1	0.91 (0.52–1.61)	0.86 (0.50–1.49)
Intracerebral hemorrhage	Case	117	59	69	103	125
	Control	54	66	87	117	149
	Model 1	1.56 (1.16–2.11)^b^	1.08 (0.76–1.52)	1	1.06 (0.78–1.44)	1.02 (0.76–1.37)
	Model 2	1.27 (0.93–1.74)	1.05 (0.74–1.50)	1	0.94 (0.69–1.28)	0.94 (0.70–1.28)
Subarachnoid hemorrhage	Case	40	20	32	44	55
	Control	13	22	33	53	70
	Model 1	1.54 (0.96–2.45)	0.97 (0.55–1.71)	1	0.92 (0.59–1.45)	0.89 (0.58–1.38)
	Model 2	1.20 (0.73–1.97)	0.78 (0.44–1.39)	1	0.95 (0.59–1.52)	0.90 (0.57–1.42)

Model 1, matched for age; Model 2, Model 1 plus adjusted for body mass index, smoking, drinking, exercise, hypertension, hyperlipidemia and diabetes.

^a^*P* < 0.001.

^b^*P* < 0.01.

**Table 3B.** Multivariable-adjusted odds ratios and 95% confidence intervals of stroke and its subtypes according to height in men aged 60–79 years, Aichi, Japan

		Height, cm				

		≤160.9	161.0–164.9	165.0–167.9	168.0–170.9	≥171.0
Total stroke	Case	553	284	267	230	171
	Control	417	309	306	269	204
	Model 1	1.23 (1.06–1.43)^b^	1.03 (0.87–1.22)	1	0.99 (0.83–1.18)	0.98 (0.81–1.18)
	Model 2	1.15 (0.99–1.33)	1.00 (0.84–1.18)	1	1.04 (0.87–1.24)	0.95 (0.79–1.16)
Thrombotic stroke	Case	293	165	128	101	79
	Control	212	148	171	134	101
	Model 1	1.37 (1.11–1.69)^b^	1.24 (0.98–1.56)	1	1.01 (0.78–1.31)	1.02 (0.77–1.36)
	Model 2	1.23 (1.00–1.52)	1.07 (0.85–1.35)	1	0.95 (0.73–1.24)	0.89 (0.67–1.18)
Embolic stroke	Case	58	38	41	49	44
	Control	59	51	38	45	37
	Model 1	0.95 (0.64–1.43)	0.82 (0.53–1.28)	1	1.00 (0.66–1.52)	1.05 (0.68–1.61)
	Model 2	1.04 (0.69–1.57)	0.98 (0.62–1.55)	1	1.17 (0.76–1.79)	1.15 (0.74–1.77)
Intracerebral hemorrhage	Case	165	69	80	65	43
	Control	123	89	84	77	49
	Model 1	1.18 (0.90–1.55)	0.90 (0.65–1.24)	1	0.94 (0.68–1.30)	0.96 (0.66–1.39)
	Model 2	1.12 (0.85–1.48)	1.03 (0.74–1.43)	1	1.16 (0.82–1.62)	1.08 (0.74–1.58)
Subarachnoid hemorrhage	Case	37	12	18	15	5
	Control	23	21	13	13	17
	Model 1	1.09 (0.62–1.93)	0.62 (0.30–1.29)	1	0.92 (0.46–1.84)	0.38 (0.14–1.03)
	Model 2	1.05 (0.57–1.94)	0.81 (0.37–1.77)	1	0.97 (0.47–2.02)	0.51 (0.18–1.47)

Model 1, matched for age; Model 2, Model 1 plus adjusted for body mass index, smoking, drinking, exercise, hypertension, hyperlipidemia and diabetes.

^b^*P* < 0.01.

In contrast to men, compared to those with 152.3–155.1 cm height, women with height less than 150.0 cm (shortest quintile) did not have higher risks of total stroke and any stroke subtypes in both younger and older women (Table 4A and Table 4B). On the contrary, the tallest height quintile was significantly associated with increased risks of total stroke and intracerebral hemorrhage in older women (model 2 ORs 1.25 and 1.44, respectively), and continuous height tended to be positively associated with the risks of these strokes (Figure 1B). The association between height and intracerebral hemorrhage seemed to differ by age group (*P* for interaction of age group with intracerebral hemorrhage = 0.064).

**Table 4A.** Multivariable-adjusted odds ratios and 95% confidence intervals of stroke and its subtypes according to height in women aged 40–59 years, Aichi, Japan

		Height, cm				

		≤149.9	150.0–152.2	152.3–155.1	155.2–158.1	≥158.2
Total stroke	Case	168	192	228	194	294
	Control	112	203	214	222	325
	Model 1	1.17 (0.96–1.43)	0.94 (0.78–1.14)	1	0.90 (0.75–1.09)	0.92 (0.77–1.09)
	Model 2	1.12 (0.91–1.37)	0.98 (0.80–1.18)	1	0.98 (0.81–1.19)	0.99 (0.83–1.18)
Thrombotic stroke	Case	57	79	69	70	102
	Control	40	71	78	95	93
	Model 1	1.26 (0.88–1.78)	1.12 (0.81–1.55)	1	0.90 (0.65–1.26)	1.11 (0.82–1.51)
	Model 2	1.17 (0.82–1.68)	1.14 (0.82–1.58)	1	0.98 (0.70–1.38)	1.10 (0.80–1.51)
Embolic stroke	Case	18	11	19	10	28
	Control	12	13	16	18	27
	Model 1	1.11 (0.58–2.13)	0.84 (0.40–1.78)	1	0.65 (0.30–1.41)	0.92 (0.50–1.71)
	Model 2	1.11 (0.55–2.23)	1.08 (0.48–2.44)	1	0.83 (0.36–1.90)	1.06 (0.55–2.05)
Intracerebral hemorrhage	Case	62	58	73	56	86
	Control	34	68	70	65	98
	Model 1	1.27 (0.90–1.78)	0.90 (0.64–1.27)	1	0.91 (0.64–1.29)	0.91 (0.67–1.25)
	Model 2	1.14 (0.80–1.61)	0.82 (0.58–1.17)	1	0.96 (0.67–1.36)	0.95 (0.69–1.31)
Subarachnoid hemorrhage	Case	31	44	67	58	78
	Control	26	51	50	44	107
	Model 1	0.96 (0.62–1.47)	0.81 (0.55–1.19)	1	0.99 (0.70–1.41)	0.73 (0.53–1.02)
	Model 2	1.02 (0.66–1.58)	0.96 (0.65–1.41)	1	1.00 (0.70–1.43)	0.90 (0.64–1.26)

Model 1, matched for age; Model 2, Model 1 plus adjusted for body mass index, smoking, drinking, exercise, hypertension, hyperlipidemia and diabetes.

**Table 4B.** Multivariable-adjusted odds ratios and 95% confidence intervals of stroke and its subtypes according to height in women aged 60–79 years, Aichi, Japan

		Height, cm				

		≤149.9	150.0–152.2	152.3–155.1	155.2–158.1	≥158.2
Total stroke	Case	422	324	265	176	263
	Control	413	359	294	237	147
	Model 1	1.06 (0.91–1.24)	1.00 (0.85–1.18)	1	0.90 (0.74–1.09)	1.36 (1.14–1.61)^a^
	Model 2	1.04 (0.89–1.21)	0.98 (0.83–1.15)	1	0.98 (0.81–1.19)	1.25 (1.05–1.49)^c^
Thrombotic stroke	Case	195	138	119	65	114
	Control	178	153	115	106	79
	Model 1	1.03 (0.82–1.29)	0.93 (0.73–1.19)	1	0.75 (0.55–1.01)	1.16 (0.90–1.50)
	Model 2	1.02 (0.81–1.29)	0.96 (0.75–1.23)	1	0.82 (0.61–1.11)	1.12 (0.86–1.45)
Embolic stroke	Case	62	47	37	26	42
	Control	67	60	41	35	11
	Model 1	1.01 (0.67–1.52)	0.92 (0.60–1.42)	1	0.91 (0.55–1.50)	1.70 (1.09–2.66)^c^
	Model 2	1.02 (0.68–1.55)	0.98 (0.64–1.52)	1	1.03 (0.62–1.71)	1.53 (0.97–2.41)
Intracerebral hemorrhage	Case	118	85	66	54	70
	Control	111	92	99	52	39
	Model 1	1.29 (0.95–1.74)	1.20 (0.87–1.66)	1	1.27 (0.89–1.83)	1.61 (1.15–2.25)^b^
	Model 2	1.13 (0.83–1.54)	1.03 (0.74–1.43)	1	1.36 (0.95–1.96)	1.44 (1.02–2.05)^c^
Subarachnoid hemorrhage	Case	47	54	43	31	37
	Control	57	54	39	44	18
	Model 1	0.86 (0.57–1.30)	0.95 (0.64–1.42)	1	0.79 (0.50–1.25)	1.28 (0.83–1.99)
	Model 2	0.97 (0.64–1.48)	0.96 (0.64–1.46)	1	0.87 (0.55–1.40)	1.07 (0.68–1.70)

Model 1, matched for age; Model 2, Model 1 plus adjusted for body mass index, smoking, drinking, exercise, hypertension, hyperlipidemia and diabetes.

^a^*P* < 0.001.

^b^*P* < 0.01.

^c^*P* < 0.05.

We obtained essentially similar results in both sensitivity analyses (data not shown).

## DISCUSSION

This is the first report that examined the associations between adult height and ischemic stroke subtypes. We found that height was significantly inversely associated with the risks of total and thrombotic strokes in men regardless of age group. We also found that height was significantly inversely associated with the risks of embolic stroke and intracerebral hemorrhage in younger men. In contrast, height did not seem to be associated with total stroke and any stroke subtypes in younger women. Moreover, the tallest height quintile was significantly associated with increased risks of total stroke and intracerebral hemorrhage in older women.

The findings in men are consistent with those in previous literature regarding stroke incidence or mortality. Although there existed certain sex and age group differences, one of the possible explanations for the inverse relationships would be the effect of height on hemodynamics. Short height possibly induces shortened return time for reflected waves, augmented systolic waves, and faster heart rates. These changes could stiffen the aorta, increase left ventricle workload, and shorten diastole, which would elevate central aortic pressure^22^ and then, the risk of stroke.^23^

Another potential explanation is related to differences in the socioeconomic or nutritional statuses during childhood according to height.^24^ Early life malnutrition or smaller growth has also been associated with suboptimal vascular or hemostatic factors.^25^^,^^26^ In addition, socioeconomic position in childhood has been associated with the risk of stroke.^27^^,^^28^

Other explanations would be the association between shorter stature and fewer glomeruli,^29^ which is related to high blood pressure.^25^^,^^30^^,^^31^ However, since we did not observe higher prevalence of hypertension among shorter men and women, as well as that the present findings did not alter after adjustment for hypertension, this possibility remains highly speculative.

Interestingly, we found that the tallest height quintile was significantly positively associated with incidence of total stroke and intracerebral hemorrhage in older women. Similar association was also found for embolic stroke. This finding has not been made in previous studies. One plausible pathway, especially for embolic stroke, might be Af, which is the most common cause of cardioembolic stroke,^32^ as height was positively associated with Af incidence in older people.^33^^,^^34^ Af-associated stroke was typically frequent among older people and more often among older women.^35^ However, we observed significant inverse association in men aged 40–59 years in contrast to the insignificant positive association in women aged 60–79 years. Although there are other origins of embolus, it would be difficult to explain the inverse association observed in men aged 40–59 years from a viewpoint of Af being the mediator between height and embolic stroke. Unfortunately, we had no data on embolus origin, such as Af, either in the Aichi Prefecture Cardiovascular Disease Registry or in the controls. Further studies should be done collecting both the presence of Af and the use of anticoagulation drug therapy.

This study has several limitations that warrant discussion. First, methods for obtaining height differed between cases and controls. Although height of cases was registered by doctors, the registration form did not specify how the height should be obtained. Our survey indicated that height of cases had been likely to be reported. Since the proportions of self-report differ according to stroke subtypes or age group, it might have brought unknown bias in the present study. However, the proportions of self-report in non-thrombotic stroke or in older patients did not differ between men and women, which did not strongly indicate that the association observed in older women was due to the bias. Second, we do not have information on the socioeconomic and nutritional statuses of cases and controls during childhood, and these might have confounded the associations. Third, childhood height was not available in the present study. A recent report from Danish study showed that short height at 7 to 13 years was significantly associated with increased risks of ischemic stroke in both men and women, and with intracerebral hemorrhage in men, while growth during this period was not,^36^ suggesting that childhood height might be more influential in stroke incidence than adult height.

Strengths of the present study include a large number of stroke cases and the use of ischemic stroke subtypes as the outcome. Although we used population-based controls, sampling period differed slightly between cases (from 2001 through 2009) and controls (in 2001, 2004, 2009, and 2012).

In conclusion, we reported the associations between adult height and ischemic stroke subtypes for the first time. Continuous height was linearly and inversely associated with the risks of total and thrombotic strokes, and the shortest height quintile compared to the reference group was associated with increased risks of these strokes in both younger and older men. Continuous height was linearly and inversely associated with the risks of embolic stroke and intracerebral hemorrhage in younger men, but the associations between the shortest height quintile and these stroke risks were not statistically significant. There did not seem any apparent associations between height and total stroke or any stroke subtypes in younger women. In contrast, continuous height tended to be positively associated with the risks of total stroke and intracerebral hemorrhage, and the tallest height quintile compared to the reference group showed statistically significantly increased risks of these strokes in older women. Reasons for these inconsistent findings need to be elucidated in future studies.

## SUPPLEMENTARY MATERIAL

eMaterials 1. Supplementary methods

eTable 1. Age-adjusted risk factors for stroke in total stroke cases and controls according to height in men, Aichi, Japan

eTable 2. Age-adjusted risk factors for stroke in total stroke cases and controls according to height in women, Aichi, Japan
