# Supplementary material for: The Association Between Adult Height and Stroke Incidence in Japanese Men and Women: A Population-based Case-Control Study
Source: J Epidemiol. 2023 Jan 5;33(1):23–30. doi: 10.2188/jea.JE20200531 (PMC9727208; doi:10.2188/jea.JE20200531)
Supplement: Supplementary file 1 [file je-33-023-s001.pdf]

eMaterials 1. Supplementary methods

The registration form did not specify how height and weight should be obtained. Therefore, we have conducted an interview survey to physicians in stroke care, and obtained a consensus that physicians would transcribe height and weight from the patient chart filled in by nurses. We then performed a further survey regarding how nurses obtain the data (Fujita Health University Ethics Review Committee approval number: HM20-402).

Among a series of cases in two wards for the care of stroke patients in Fujita Health University Hospital (n=61), we found that the proportions of cases whose height was self-reported by the patient itself were 45.2% (14/31) in men and 46.7% (14/30) in women. The proportions of cases whose height was reported by the spouse or the child were 28.3% and 6.3%, respectively. The proportion of cases whose height was measured using a plastic scale was 13.1%.

The proportions of self-report were higher in thrombotic stroke than in the other types of stroke in both men and women (57.9% vs. 25.0% in men and 66.7% vs. 26.7% in women). Similarly, the proportions were higher in stroke cases aged younger than 60 years than in stroke cases aged 60 years or older (60.0% vs. 42.3% in men and 83.3% vs. 37.5% in women).

Weight was measured on scales periodically during the admission.

eTable 1. Age-adjusted risk factors for stroke in total stroke cases and controls according to height in men, Aichi, Japan

	Height, cm					
	≤160.9	161.0–164.9	165.0–167.9	168.0–170.9	≥171.0	*P*-value
40–59 years, Cases	n=368	n=178	n=239	n=318	n=395	
Age, years	52.0	51.9	50.9	50.7	49.6	<0.001
Weight, kg	55.8	63.9	67.2	70.8	74.4	<0.001
Body mass index, kg/m^2	23.4	24.2	24.5	24.8	24.3	<0.001
Current smoking, %	30.4	54.2	51.0	57.6	57.3	<0.001
Current drinking, %	30.7	52.6	50.6	52.3	56.0	<0.001
Exercise +, %	18.3	17.5	15.1	12.9	11.3	0.062
Hypertension, %	57.4	55.8	62.8	55.1	53.7	0.243
Hyperlipidemia, %	18.4	18.4	21.3	23.3	17.6	0.321
Diabetes, %	20.2	24.9	26.4	21.6	18.7	0.145
40–59 years, Controls	n=150	n=236	n=282	n=365	n=465	
Age, years	52.6	52.2	51.5	50.4	50.0	<0.001
Weight, kg	58.3	61.8	64.6	66.8	70.9	<0.001
Body mass index, kg/m^2	23.3	23.3	23.5	23.3	23.2	0.839
Current smoking, %	36.0	34.7	35.4	31.0	35.7	0.631
Current drinking, %	53.1	61.7	65.1	61.4	66.7	<0.05
Exercise +, %	25.4	27.8	26.0	25.4	31.5	0.298
Hypertension, %	13.5	17.6	14.1	18.3	15.2	0.442
Hyperlipidemia, %	9.3	10.5	13.2	11.3	10.9	0.772
Diabetes, %	5.6	7.3	6.9	7.2	4.9	0.602
60–79 years, Cases	n=553	n=284	n=267	n=230	n=171	
Age, years	68.5	67.8	67.2	67.5	66.2	<0.001
Weight, kg	53.6	60.7	62.8	65.4	70.2	<0.001
Body mass index, kg/m^2	22.4	23.0	22.9	22.9	23.1	<0.05
Current smoking, %	28.4	40.9	35.9	38.1	38.4	<0.01
Current drinking, %	31.1	47.2	49.6	51.7	52.0	<0.001
Exercise +, %	18.5	16.2	15.4	20.9	16.6	0.494
Hypertension, %	60.9	62.7	63.5	59.1	67.3	0.490
Hyperlipidemia, %	17.4	21.1	17.6	19.1	26.8	0.080
Diabetes, %	25.6	24.3	26.9	23.0	31.4	0.379
60–79 years, Controls	n=417	n=309	n=306	n=269	n=204	
Age, years	69.5	68.0	66.7	66.8	66.3	<0.001
Weight, kg	56.7	60.6	63.5	64.9	69.7	<0.001
Body mass index, kg/m^2	22.8	22.9	23.2	22.8	23.1	0.419
Current smoking, %	22.2	21.7	20.4	21.6	18.5	0.854
Current drinking, %	50.1	52.7	58.5	58.3	64.9	<0.01
Exercise +, %	46.7	50.4	48.6	46.8	48.8	0.873
Hypertension, %	35.2	34.7	31.1	34.1	35.8	0.782
Hyperlipidemia, %	8.3	11.4	8.9	13.2	12.9	0.179
Diabetes, %	14.6	16.1	14.0	17.0	16.6	0.831

eTable 2. Age-adjusted risk factors for stroke in total stroke cases and controls according to height in women, Aichi, Japan

	Height, cm					
	≤149.9	150.0–152.2	152.3–155.1	155.2–158.1	≥158.2	*P*-value
40–59 years, Cases	n=168	n=192	n=228	n=194	n=294	
Age, years	53.5	52.9	52.8	52.0	51.5	<0.001
Weight, kg	49.6	53.7	55.3	55.9	60.7	<0.001
Body mass index, kg/m^2	23.4	23.6	23.3	22.7	22.8	0.081
Current smoking, %	14.4	19.8	22.9	32.9	31.5	<0.001
Current drinking, %	12.1	23.0	19.8	31.4	33.5	<0.001
Exercise +, %	13.2	15.4	14.8	16.7	16.7	0.866
Hypertension, %	52.4	60.4	53.6	48.9	53.0	0.239
Hyperlipidemia, %	20.1	12.7	15.5	22.0	17.0	0.123
Diabetes, %	17.7	17.9	15.5	15.3	14.9	0.877
40–59 years, Controls	n=112	n=203	n=214	n=222	n=325	
Age, years	54.8	53.1	52.7	52.1	50.3	<0.001
Weight, kg	48.6	51.1	52.8	54.2	56.7	<0.001
Body mass index, kg/m^2	22.6	22.5	22.2	22.0	21.6	<0.01
Current smoking, %	5.7	8.0	9.9	6.3	10.2	0.374
Current drinking, %	20.2	22.7	25.1	30.6	33.7	<0.05
Exercise +, %	28.1	31.7	34.1	36.1	34.8	0.631
Hypertension, %	5.2	15.4	13.8	13.1	13.0	0.119
Hyperlipidemia, %	14.6	6.4	7.2	9.0	7.5	0.104
Diabetes, %	3.7	2.2	1.7	3.6	3.3	0.680
60–79 years, Cases	n=422	n=324	n=265	n=176	n=263	
Age, years	69.0	68.6	67.4	67.0	67.0	<0.001
Weight, kg	47.7	52.4	54.7	56.6	60.6	<0.001
Body mass index, kg/m^2	22.7	23.0	23.1	23.0	22.6	0.512
Current smoking, %	7.7	11.1	14.0	14.2	27.6	<0.001
Current drinking, %	6.1	12.2	12.7	16.8	29.8	<0.001
Exercise +, %	15.2	17.0	16.6	19.3	22.4	0.174
Hypertension, %	61.9	62.9	65.8	57.0	55.7	0.115
Hyperlipidemia, %	26.1	21.3	29.0	25.5	21.6	0.165
Diabetes, %	23.6	21.8	18.3	18.4	18.3	0.320
60–79 years, Controls	n=413	n=359	n=294	n=237	n=147	
Age, years	69.4	68.0	67.3	66.7	65.6	<0.001
Weight, kg	48.0	51.7	52.9	53.8	56.2	<0.001
Body mass index, kg/m^2	22.5	22.7	22.3	21.9	21.7	<0.01
Current smoking, %	3.8	4.5	5.0	3.9	4.2	0.954
Current drinking, %	12.4	14.0	15.1	14.0	21.7	0.106
Exercise +, %	47.3	43.1	45.5	48.1	52.6	0.366
Hypertension, %	39.3	33.8	32.0	25.7	28.8	<0.01
Hyperlipidemia, %	12.6	12.0	18.7	14.8	21.7	<0.05
Diabetes, %	8.7	8.6	7.2	6.9	4.5	0.518
